# Integrated environmental and nutritional efficiencies of local foods in Indonesia: a cradle-to-grave enviro-nutritional life cycle assessment

**DOI:** 10.1016/j.lanwpc.2026.101977

**Published:** 2026-09-09

**Authors:** Sarah J. McLaren, Graham A. McAuliffe, Thomas C. Ponsioen, Ratna C. Purwestri, Flaminia Ortenzi, Ty Beal

**Affiliations:** a New Zealand Life Cycle Management Centre, Massey University, Palmerston North, New Zealand; b Harper Food Innovation, Harper Adams University, Newport, UK; c FootPrinting, Wageningen, Netherlands; d Department of Nutrition, Universitas Brawijaya, Malang, Indonesia; e Faculty of Forestry and Wood Sciences, Czech University of Life Sciences Prague, Czech Republic; f Global Alliance for Improved Nutrition, Geneva, Switzerland; g Global Alliance for Improved Nutrition, Washington, DC, USA

**Keywords:** Nutritional life cycle assessment (nLCA), Indonesia, Nutritional Value Score (NVS), Enviro-nutritional efficiency, Sustainable food systems

## Abstract

**Background:**

Indonesia faces simultaneous pressure to improve nutrition and reduce the environmental burden of food systems yet is underrepresented in nutritional life cycle assessment (nLCA) literature. Most existing nutritional functional units (nFUs) were developed in high-income countries, with no consensus on which nFU suits which purpose. Food and Agriculture Organization guidance sets out how such choices should be justified against a study's goal and scope. We evaluated the enviro-nutritional efficiencies of commonly consumed Indonesian foods using a holistic nFU.

**Methods:**

We modelled 90 Indonesian foods across five food groups (animal-source foods; pulses, nuts, and seeds; starchy staples; vegetables; and fruits) from cradle-to-grave, including home and street preparation, and waste management. LCA results for seven environmental impact categories were integrated with the Nutritional Value Score (NVS), a holistic food rating system capturing nutrient density and protective factors against noncommunicable diseases, using a nFU of 100 NVS. Economic allocation was applied, with cut-off for select co-products.

**Findings:**

Variability in enviro-nutritional efficiencies reached 211 times the lowest value within food groups. Animal-source foods and vegetables showed the greatest variability across most indicators; *pulses, nuts, and seeds* and fruits were most variable for water use. Primary production contributed most to environmental impacts for most foods, with exceptions for some processed foods, co-products, and fruits with high refuse. Alignment of enviro-nutritional efficiency with nutritional value was strongest for starchy staples (R^2^ = 0.65) and weakest for animal-source foods (R^2^ = 0.00).

**Interpretation:**

Combining holistic nutrient profiling with LCA can identify context-appropriate foods that deliver high nutritional value with lower environmental impact in underrepresented middle-income settings. Findings should be complemented by modelling multi-ingredient foods and sub-national heterogeneity.

**Funding:**

Nourishing Food Pathways programme.

**Research in context**

**Evidence before this study**

Previous reviews of nutritional life cycle assessment (nLCA) have documented the absence of consensus on nutritional functional units (nFUs) and a concentration of published studies in high-income countries, with limited coverage of Southeast Asian diets and food systems. Existing nutrient profiling systems applied in nLCA, predominantly variants of the Nutrient Rich Foods index family, were developed with data from the United States and do not adequately capture the complexities of the nutritional value of foods, particularly in low- and middle-income countries. The Nutritional Value Score (NVS), recently developed to address these limitations, has not previously been applied in LCA. Life cycle inventory data for Indonesian foods—particularly data reflecting local cooking practices (eg, liquefied petroleum gas stove use) and waste treatment—are scarce, and existing global meta-analyses rely heavily on proxy data from other countries. To our knowledge, no prior study has integrated LCA with a holistic food rating system to evaluate the enviro-nutritional performance of commonly consumed foods in Indonesia.

**Added value of this study**

This study is among the first to integrate LCA with a holistic nutrient profiling system—the NVS, which captures both essential nutrient density and protective factors against noncommunicable diseases—in an underrepresented upper middle-income country setting. By applying an NVS-based nutritional functional unit to 90 commonly consumed Indonesian foods across five food groups, and by modelling cradle-to-grave impacts across seven environmental categories, we generated food-level enviro-nutritional efficiencies that reflect local production, processing, preparation, and waste management practices. We also produced open-access life cycle inventory data for Indonesian foods, addressing a substantial data gap for Southeast Asia. Our results demonstrate that enviro-nutritional efficiencies vary by up to 211 times within food groups, and that the relationship between nutritional value and enviro-nutritional performance differs markedly across food groups—strongest for starchy staples and weakest for animal-source foods.

**Implications of all the available evidence**

Combined with previous work, our findings support the use of holistic nutrient profiling in environmental impact assessments to identify foods that simultaneously advance nutrition and environmental goals. For Indonesian food systems policy, findings indicate opportunities to promote nutritious foods within recommended food groups with relatively low environmental footprints such as dark green leafy vegetables, chayote, carrots, papaya, guava, cantaloupe, milk, yogurt, chicken organs, whole grains, and soy products, alongside improvements in food preparation and waste management, alternative sourcing, and supply-chain efficiency. For the broader field, the approach can be adapted to other countries and populations by localising food composition and environmental impact data, adjusting NVS component weightings to reflect specific nutritional priorities, and expanding to multi-ingredient foods. Continued investment in context-specific life cycle inventory data, water and land use characterisation factors, and sub-nationally disaggregated information on livestock management, transport, and food losses is essential to support evidence-based food systems transitions in low- and middle-income countries.

## Introduction

Indonesia has recently experienced rapid economic growth, gaining classification as an upper middle-income country and ranking as the sixteenth largest economy globally.^1^^,^^2^ However, high poverty rates (9.4%) persist, unevenly distributed across islands and urban-rural areas.^3^ This economic disparity is reflected in food systems, with significant variation in access to nutritious food and dietary intakes across regions and income groups.^4^ Protection of natural ecosystems, reduced greenhouse gas emissions, and improved nutrition are key priorities for Indonesian policymakers.^5^ These priorities are inextricably linked: land use change and climate vulnerability actively contribute to increased food insecurity and poverty, which are causally linked to malnutrition.^6^^,^^7^

To support decision-making that simultaneously addresses environmental protection and access to healthy diets, environmental impact assessment of foods should be undertaken through the lens of their nutritional contributions.^8^ However, countries such as Indonesia are underrepresented in extant literature and databases on the environmental impacts of foods, and the scarcity of local data often forces reliance on global or regional averages that do not accurately reflect local agroecological conditions or production practices.^9^ Moreover, there is no consensus on defining nutritional functional units (nFUs), and development of novel nFUs has been largely restricted to high-income countries.^10^ The Food and Agriculture Organization of the United Nations (FAO) has synthesised the opportunities and challenges of integrating environment and nutrition in LCA, recommending that the choice of nFU be justified transparently against the goal and scope of the study, that nutrient density be represented as comprehensively as the data allow, and that both beneficial and limiting nutrients be considered.^11^ That synthesis does not resolve the absence of a single agreed nFU but instead sets out the challenges to defining a standardised approach, and we follow its recommendations in framing and justifying the selection of the nFU used here.

This study evaluates the environmental impacts per unit of nutritional value (hereafter “enviro-nutritional efficiencies”) of locally available foods commonly consumed in Indonesia across five food groups. Life Cycle Assessment (LCA) is used to analyse seven environmental impact categories from cradle-to-grave. Holistic food rating systems—those that score foods across a diversity of nutrients and dietary factors relevant to both nutrient adequacy and protection against noncommunicable diseases, rather than a restricted set of nutrients—are well suited to this purpose. We use the Nutritional Value Score (NVS) to holistically assess nutritional value,^12^ integrating LCA results with the NVS using an nFU (100 NVS). This research is one of the first such studies for an underrepresented country.^13^^,^^14^ By evaluating diverse environmental impacts in terms of nutritional value and including home and street food preparation and waste management, our research offers a nuanced understanding of enviro-nutritional synergies and trade-offs across a variety of Indonesian foods.

## Methods

### Goal, scope, and functional unit

We assessed the enviro-nutritional performance of 90 commonly consumed foods in Indonesia by integrating LCA with the NVS.^12^ Foods were selected to represent five food groups—animal-source foods; pulses, nuts, and seeds; starchy staples; vegetables; and fruits—based on consumption patterns in Indonesia and the availability of LCA data. Processed foods were limited to dairy products, roasted nuts, soy products, and noodles and pasta, and congee was the only multi-ingredient food included. The functional unit was defined as 100 NVS (ie, the highest nutritional value on a scale of 1–100) of food as consumed (edible portion).^12^ For each product, the reference flow was the quantity of food purchased required to deliver 100 NVS, accounting for removal of inedible fractions (refuse), mass changes during cooking (cooking yield, including water gain or loss), and consumer waste. All reported impacts per 100 NVS therefore incorporate upstream supply chain burdens associated with the purchased quantity needed to deliver the consumed portion. A secondary goal was to generate life cycle inventory (LCI) data for Indonesia, an upper middle-income country currently underrepresented in the LCA literature; full inventory data are available in an accompanying open-access dataset.^15^

### System boundaries

System boundaries were cradle-to-grave, covering primary production, post-harvest handling, processing (where applicable), primary packaging, distribution (ambient, cooled, or frozen), retail, the user stage (storage and preparation), and waste treatment, including food losses throughout the life cycle. Secondary and tertiary packaging were excluded. Where cooking was required, it was assumed to occur at home—with the exception of congee, assumed to be prepared as street food—reflecting the central role of home-cooked meals in Indonesian daily life. Cooking yields and refuse fractions for each food item were sourced from USDA FoodData Central and FAO commodity tables and incorporated into the inventory dataset.^15^ Supply-chain losses for post-harvest handling, processing, retail, and transport were additionally applied as food group-specific estimates based on Southeast Asian averages from FAO.^16^ These loss factors reflect average commodity-level losses prior to consumption and are conceptually distinct from household refuse and cooking yield adjustments.

### Nutritional inventory and impact assessment

Nutritional value was assessed using the NVS, a nutrient profiling system scaled from 1 (lowest) to 100 (highest) that captures both essential nutrient density and protective factors against noncommunicable diseases.^12^ The NVS is the weighted mean of seven normalised dietary attribute scores—vitamins (20%), minerals (20%), protein (12.5%), n-3 fatty acids (10%), fibre (7.5%), Calories (7.5%), and nutrient ratios comprising sodium:potassium, saturated:unsaturated fat, and carbohydrate:fibre (22.5%)—with a 25% penalty applied to ultraprocessed foods before normalisation. Ultraprocessed foods were identified using the Nova classification; traditional Indonesian fermented and processed foods such as tempeh and tofu are not classified as ultraprocessed and therefore incur no penalty. Scores are calculated per unit energy and per unit mass, and averaged, to ensure that foods are not unduly favoured or penalised for having low or high Calorie density. This dual normalisation also underpins the comparability of a fixed nutritional functional unit across food groups that differ substantially in moisture and energy density. Food composition values for Indonesian foods were compiled using a combination of USDA FoodData Central and national and regional food composition tables, following the approach described in detail elsewhere.^12^ The NVS captures protection against noncommunicable diseases through nutrients and dietary factors that are predictive of noncommunicable disease risk—including the sodium:potassium, saturated:unsaturated fat, and carbohydrate:fibre ratios, the fibre and Calories scores, and the n-3 fatty acids score, together with the ultraprocessing penalty—rather than by modelling disease incidence or clinical outcomes directly.^12^ In its development, the NVS showed moderate convergent validity with Nutri-Score (R = 0.58) and the Health Star Rating (R = 0.63), and deliberately weak agreement for ultraprocessed foods.^12^

### Environmental inventory analysis

Primary production locations were identified from national production and import statistics for 2016–2021, as detailed in the accompanying dataset.^15^ Where a food was produced domestically, Indonesia was modelled as the country of origin; where it was predominantly imported, the single largest country of origin was modelled. For foods with two major suppliers (eg, beef from Australia and India; pork from Europe and the USA), the item was modelled as two separate foods. For foods produced or imported by Indonesia but absent from FAOSTAT statistics, including all vegetables and a subset of foods in other groups, we assumed domestic production and used a proxy dataset from the country considered closest to the likely Indonesian situation, on the recommendation of an Indonesian scientist in the authorship team.

Unit processes for primary production and processing were drawn from four background databases: Agri-footprint 6.3, ecoinvent 3.9.1, Agribalyse 3.1, and the World Food LCA Database (WFLDB) 3.5.^17^, ^18^, ^19^ Selection of data sources prioritised geographical representativeness (country-specific > regional proxy > extra-regional proxy) and technological representativeness (precise crop or animal species > similar proxy). Where no suitable proxy existed, processes were constructed from national and international reports and peer-reviewed literature, combined with parameters from the best available proxy datasets and tailored emission modelling. Country-specific flows were adapted for Indonesia where relevant, most notably water flows and the electricity grid mix. Transport processes were selected from ecoinvent 3.9. Storage (retail and home) and home cooking processes were selected from WFLDB 3.5 and adapted for Indonesia, with cooking modelled as 100% liquefied petroleum gas stove use to reflect a distinctive feature of Indonesian preparation practices, particularly in lower-density areas. Full technical details of the inventory, including food-group-specific supply-chain losses, are provided in the accompanying dataset.^15^

### Multifunctionality and allocation

Economic allocation was applied to multifunctional processes as standard, with cut-off (ie, zero allocation) used where co-products have no or negligible economic value at harvest.^20^ This was applied to drumstick, pumpkin, and sweet potato leaves; chicken meat from egg-production systems (100% allocated to eggs); and tree fern leaves, which grow naturally. For fish, all impacts were allocated to fish meat and none to co-products such as fish oil and meal, because fish is the core product of Indonesian fisheries.

### Environmental impact assessment

Life cycle impact assessment was conducted using midpoint indicators, consistent with the majority of nLCA studies and enabling comparability with existing food LCA literature.^10^ Seven impact categories were assessed: climate change (GWP100), water use (AWARE-based scarcity-weighted water consumption),^21^ marine eutrophication, freshwater eutrophication, terrestrial acidification, particulate matter formation, and land use (expressed as m^2^ × year crop equivalents). Full method citations and characterisation factors are provided in the accompanying dataset.^15^ Acidification was expressed in kg SO_2_-eq, consistent with common practice in agri-food LCA.^10^ Endpoint indicators were not used because endpoint cause-effect modelling adds uncertainty.^22^ Fossil resource use was excluded to keep the assessment focused on impacts most directly relevant to the study scope. Toxicity categories were excluded because of large uncertainties in toxicity characterisation factors.^22^ Ozone depletion, ionising radiation, photochemical ozone formation, and mineral and metal resource use were omitted because agri-food systems contribute only a small fraction of these impacts.^22^ To support interpretation, we also calculated a single score using the Environmental Footprint 3.1 method^23^ to summarise environmental impact across categories overall and within each food group; however, single-score results should be interpreted cautiously because of subjectivity in the normalisation and weighting steps.^22^ The single score was obtained by normalising each Environmental Footprint impact category result against the corresponding EF3.1 global per-capita normalisation reference and combining the normalised results using the EF3.1 category weights; the normalisation references and weights applied are reported in the accompanying dataset.^15^ As the EF3.1 single score is defined across the complete Environmental Footprint category set, it also incorporates categories that are not reported individually here, including the highly uncertain results of the ecotoxicity and human toxicity categories; it is therefore interpreted with caution and not as a headline result.

### Data quality

Data quality was assessed using a pedigree-matrix approach adapted from the European Commission's Product Environmental Footprint guidance.^24^ Five indicators were considered—reliability, completeness, temporal correlation, geographical correlation, and technological correlation—each scored on a five-point ordinal scale (1 = highest quality, 5 = lowest) and averaged to derive a primary-production rating. For downstream life-cycle stages, a single aggregated score was assigned per stage, reflecting more limited disaggregated information. An overall data quality rating for each food was calculated as the arithmetic mean of stage-level scores. This provides a qualitative appraisal of data quality rather than a propagated quantification of uncertainty. The full data quality assessment accompanies the inventory dataset.^15^

### Role of the funding source

The funders had no role in study design; in the collection, analysis, or interpretation of data; in the writing of the report; or in the decision to submit the paper for publication.

## Results

### Environmental impacts across food groups

**Fig. 1:**
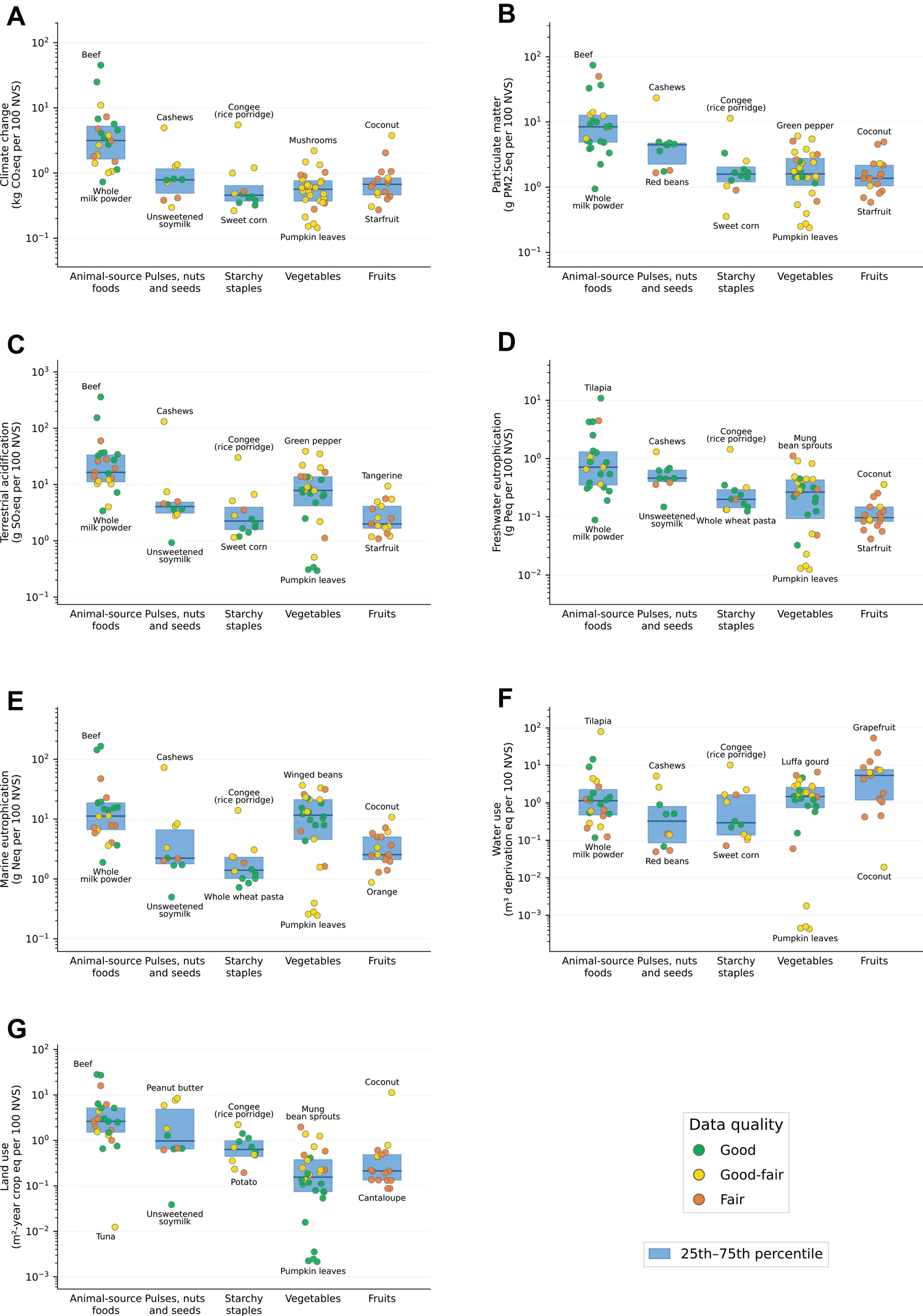
**Environmental impacts per unit of nutritional value for Indonesian foods by food group.** Environmental impact results for 90 Indonesian foods expressed per nutritional functional unit (nFU; 100 Nutritional Value Score [NVS]). Panels show results for seven impact categories: (A) climate change, (B) particulate matter formation, (C) terrestrial acidification, (D) freshwater eutrophication, (E) marine eutrophication, (F) water use, and (G) land use. Individual points represent food-level results, grouped by food category. Boxplots summarise distributions within each food group, showing medians and interquartile ranges.

The enviro-nutritional efficiencies per 100 NVS for seven environmental impact categories for individual foods and food groups are shown in Fig. 1. For climate change, particulate matter, and terrestrial acidification, most animal-source foods (25th–75th percentile) have lower enviro-nutritional efficiencies (ie, higher numeric values) than many foods in other groups. For freshwater and marine eutrophication, the ranges within food groups largely overlap. For water use, the fruit and vegetable groups have lower efficiencies due to irrigation demand, while for land use, these groups have higher efficiencies due to higher yields and, for vegetables, high NVS. Within food groups, there is a large range of efficiencies in the animal-source food and vegetable groups across all environmental indicators. The smallest variation is generally seen in the fruit group, with the notable exception of water use.

### Environmental impacts within food groups

#### Animal-source foods

**Fig. 2:**
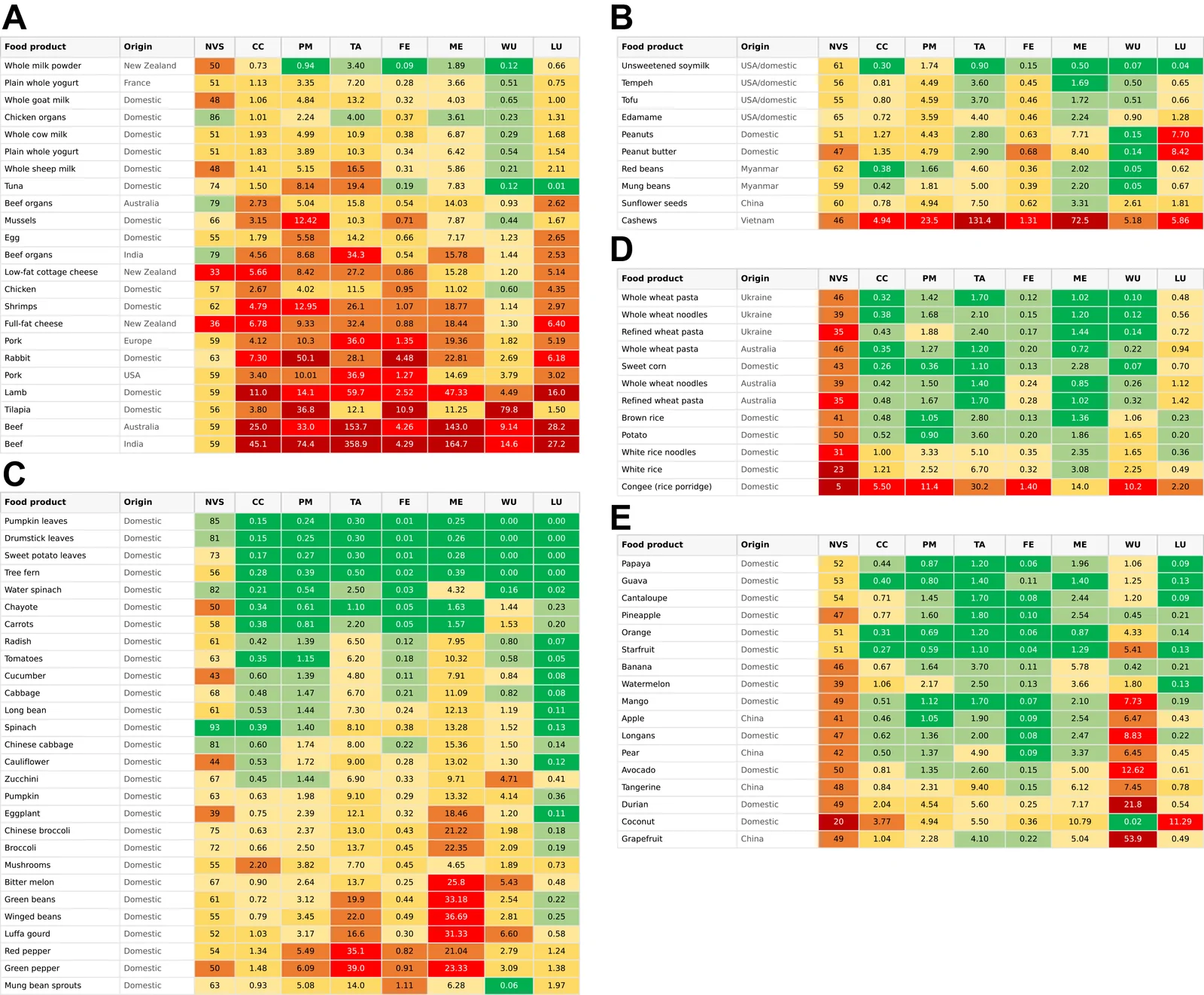
**Nutritional value scores and enviro-nutritional point estimates for Indonesian foods.** Heatmap showing NVS and corresponding environmental impact point estimates expressed per nFU (100 NVS) for 90 Indonesian foods. Panels represent (A) animal-source foods, (B) pulses, nuts, and seeds, (C) vegetables, (D) starchy staples, and (E) fruits. Rows represent individual food items and columns represent nutritional and environmental indicators. Green shading represents lower environmental impact or higher nutritional value; red shading represents higher impact or lower nutritional value.

Dairy products, except for cheese, have the highest enviro-nutritional efficiencies, while beef, lamb, rabbit, pork, and tilapia have the lowest efficiencies in this food group (Fig. 2). There is particularly high variability for water use (0.12–79.8 m^3^, with all foods except tilapia at or below 14.6 m^3^) and land use (0.01–28.2 m^2^-year crop equivalents), and high variability across other impact categories (up to 121 times the lowest result, driven by tilapia for freshwater eutrophication). NVS scores for animal-source foods range from 33 to 86.

#### Pulses, nuts, and seeds

Cashews stand out across all eight metrics (NVS plus seven environmental indicators), with the lowest efficiencies for most environmental impact categories (Fig. 2). Soybean products have the highest efficiencies. Excluding cashews, variability is low for climate change, particulate matter, terrestrial acidification, and freshwater eutrophication (3–8 times the lowest result), and higher for marine eutrophication, water use, and land use (up to 211 times the lowest result). The NVS shows low variability (46–65), meaning differences in enviro-nutritional efficiency are predominantly related to supply-chain activities.

#### Starchy staples

Congee—the only multi-ingredient food in the study—has 3–5 times lower enviro-nutritional efficiency across all categories relative to the next lowest efficiency food (Fig. 2). Aside from congee, white rice and rice noodles have the lowest efficiency, while whole wheat pasta and noodles and sweet corn have the highest. Excluding congee, water use shows the greatest within-group variability (up to ∼32 times the lowest result), driven in part by sweet corn's very low irrigation footprint; other categories range from approximately 3 to 9 times. NVS variability is very high (5–50), partly driven by the congee outlier.

#### Vegetables

The five leafy vegetables (pumpkin leaves, drumstick leaves, sweet potato leaves, tree fern, and water spinach) have the highest enviro-nutritional efficiencies in this group (Fig. 2). Excluding leafy vegetables, variability ranges from low (climate change and particulate matter, up to 10 times) to moderate (eutrophication, acidification, and land use, up to 40 times) or high (water use, more than 100 times). High NVS variability (39–93) concurrently affects the enviro-nutritional rankings.

#### Fruits

Imported fruits from China rank among the lowest-efficiency fruits, primarily driven by high water use (Fig. 2). Excluding coconut (an outlier for several categories), variability is high for water use (128 times the lowest result) and low for other categories (less than 15 times). NVS shows high variability (20–54).

### Environmental impacts by life cycle stage

**Fig. 3:**
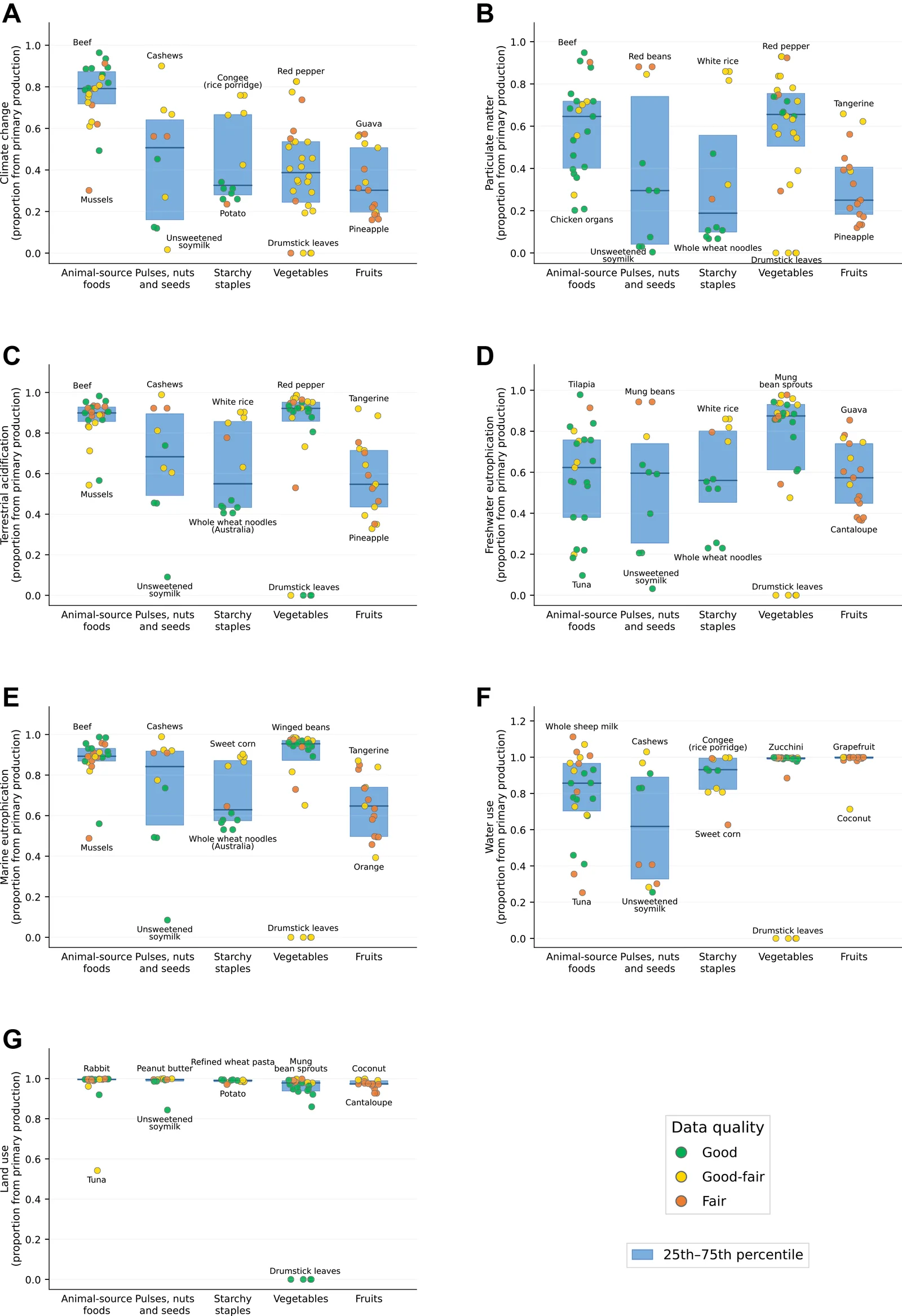
**Contribution of primary production to environmental impacts of selected Indonesian foods.** Proportion of total cradle-to-grave environmental impacts attributable to primary production for selected foods. Panels show results for seven impact categories. Total impacts include all life-cycle stages from primary production through processing, transport, preparation and cooking, and food waste streams. Panels represent (A) climate change, (B) particulate matter formation, (C) terrestrial acidification, (D) freshwater eutrophication, (E) marine eutrophication, (F) water use, and (G) land use.

Primary production alone accounts for the highest proportion of environmental impacts across most foods and indicators analysed (Fig. 3). The primary production stage contributes at least 30% of results for all impact categories across all food groups, with fruit for climate change and particulate matter as the main group-level exceptions. For land use, primary production dominates across all food groups (typically >95%), with certain dark green leafy vegetables and tuna as food-level exceptions. For terrestrial acidification, marine eutrophication, and water use, primary production contributes the majority of impacts in most food groups, with fruits (terrestrial acidification and marine eutrophication) and pulses, nuts, and seeds (water use) as the main exceptions. Notable food-level exceptions across categories include leafy vegetables with zero assigned primary production impacts as low-value co-products, and soy products with smaller impacts due to leguminous nitrogen fixation. The contribution of primary production is most variable within the pulses, nuts, and seeds group, followed by starchy staples and animal-source foods. Food-level numerical results underlying Fig. 3, for every food and impact category, are available in the accompanying open-access dataset.^15^

### Relationship between nutritional value and enviro-nutritional performance

**Fig. 4:**
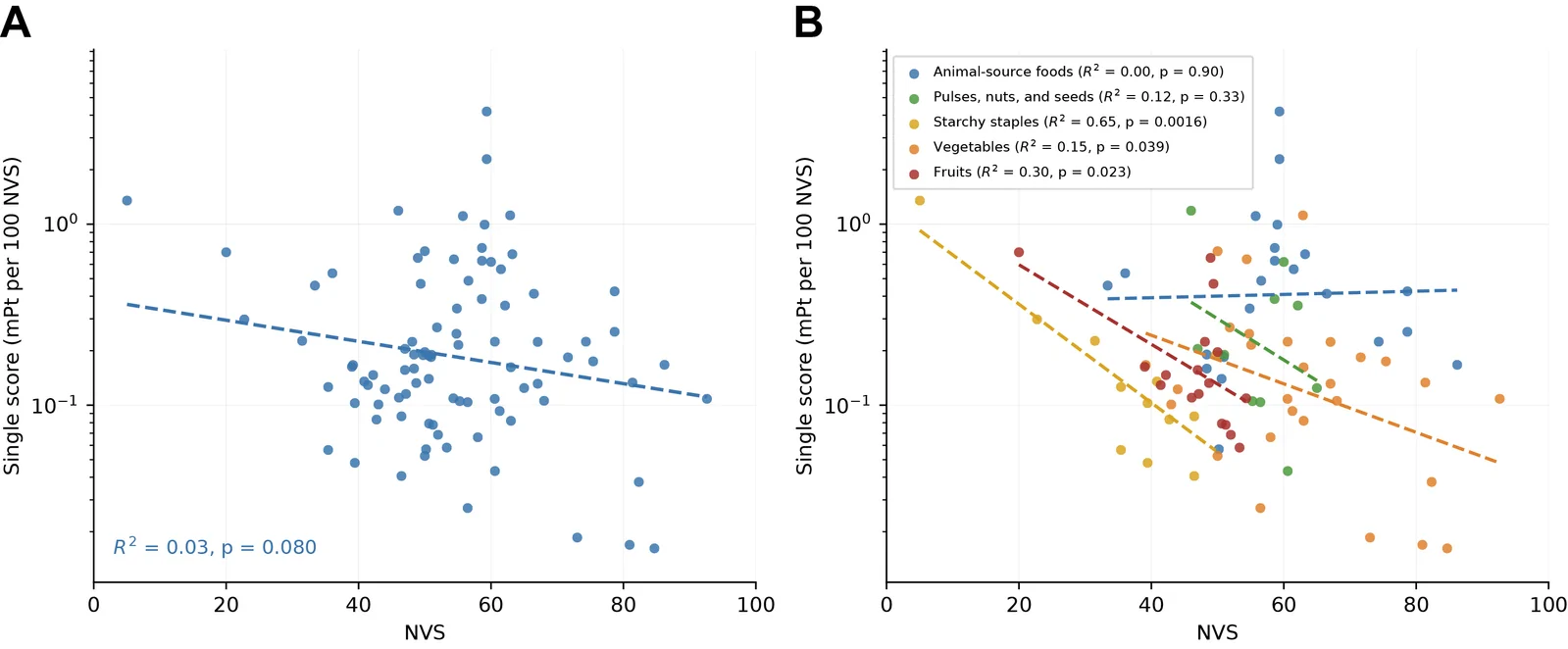
**Relationship between nutritional value and enviro-nutritional performance for Indonesian foods.** (A) Scatter plot showing the relationship between NVS and the enviro-nutritional single score across all food groups, with simple linear regression. (B) Scatter plot showing the same relationship organised by food group, with food-group-specific linear regressions and corresponding R^2^ and p values. Single scores are based on the full EF/PEF single score (Environmental Footprint 3.1 method),^23^ expressed per 100 NVS, with lower values indicating better enviro-nutritional performance. In both panels the single score is shown on a logarithmic scale, regressions are fitted to log10-transformed single scores, and regression lines are truncated to the range of the data. R^2^ values are accompanied by two-sided significance levels: overall p = 0.080; starchy staples p = 0.0016; fruits p = 0.023; vegetables p = 0.039; pulses, nuts, and seeds p = 0.33; animal-source foods p = 0.90.

The overall relationship between NVS and enviro-nutritional single score per 100 NVS across all foods is weak (R^2^ = 0.03; Fig. 4A), although some within-group patterns emerge (Fig. 4B). Foods with high NVS scores tend to have lower single scores, indicating better enviro-nutritional efficiency; however, most foods with intermediate NVS scores (40–65) do not exhibit this correlation. Across food groups, starchy staples exhibit a moderate correlation (R^2^ = 0.65), followed by fruits (R^2^ = 0.30), vegetables (R^2^ = 0.15), and pulses, nuts, and seeds (R^2^ = 0.12; Fig. 4B). The animal-source food group shows the weakest correlation (R^2^ = 0.00). Reflecting the differing number of foods per group, the relationships for starchy staples (p = 0.0016), fruits (p = 0.023), and vegetables (p = 0.039) reach conventional statistical significance, whereas the overall relationship (p = 0.080) and those for pulses, nuts, and seeds (p = 0.33) and animal-source foods (p = 0.90) do not.

## Discussion

Across 90 foods in five food groups, enviro-nutritional efficiencies varied by up to 211 times within a single food group, with animal-source foods and vegetables the most variable across most impact categories. Primary production was the dominant contributor to environmental impact for most foods, with processed foods, low-value co-products, and fruits with high refuse as the main exceptions. The strength of the relationship between nutritional value and enviro-nutritional performance differed markedly among food groups, being strongest for starchy staples and effectively absent for animal-source foods. These findings are interpreted below and placed in the context of the wider nutritional LCA literature.

The dominance of primary production as the largest contributor to environmental inefficiencies is partly due to the selection of primarily unprocessed and minimally processed foods; for processed foods such as soy products, processing is also a relevant environmental hotspot. For vegetables and fruits, distribution and transport contribute significantly due to high water content and biomass subsequently discarded during home preparation, as well as food waste.

At the primary production stage, the main impact drivers for foods with the largest footprints include low fertiliser use efficiency in cashew cultivation; high irrigation demand for grapefruit, and water evaporation from tilapia ponds (water use); phosphate losses from livestock excretion and tilapia farming (freshwater eutrophication); enteric fermentation from beef cattle and lamb (climate change); and manure from all livestock systems (marine eutrophication, particulate matter, and terrestrial acidification). At the distribution stage, the main drivers are transport mode and nutritional inefficiency. At the processing stage, fossil energy use dominates, and at the waste treatment stage, refuse generation and open dumping and burning are key drivers. This information is essential for identifying opportunities to reduce impacts through more sustainable production, distribution, processing, and waste treatment practices.

The high impacts for climate change, particulate matter, and terrestrial acidification among animal-source foods are partly due to methane and nitrous oxide emissions from manure and enteric fermentation, most notably in ruminant systems. The relatively low impacts of imported milk powder and yogurt, despite moderate NVS values, reflect environmentally efficient dairy systems in the countries of origin.^17^, ^18^, ^19^ The high impacts for beef, lamb, rabbit, and pork can partly be explained by high feed conversion ratios and, for ruminants, higher methane emissions relative to monogastric animals—demonstrating the complexities of local and global heterogeneity in livestock production systems.^25^ Higher proportions of total livestock system impacts are assigned to meat compared to co-products like offal when using economic allocation, which explains why chicken organs rank similarly to dairy products.

Placing these results in an international context, the broad patterns are consistent with the wider food LCA and nLCA literature,^26^ even though the absolute values are specific to Indonesia. The dominance of enteric fermentation and manure management in the climate change, terrestrial acidification, and particulate matter footprints of ruminant meats mirrors findings from high-income countries and other settings, reinforcing that heterogeneity in livestock systems, rather than geography alone, drives much of the variation between foods. The comparatively low impacts of cereals and other starchy staples per unit mass, and the high irrigation-driven water footprints of certain fruits and vegetables, are likewise consistent with global assessments. What is distinctive in the Indonesian case is how these patterns interact with country-specific sourcing, including fruit imported from China, near-universal liquefied petroleum gas cooking, and open dumping and burning at end of life.

The particularly high congee impacts are a consequence of its typically high salt content when sold as street food (increasing the sodium:potassium ratio penalty in the NVS), high carbohydrate:fibre ratio, and low nutrient density overall.^12^ Variability in other vegetables is explained primarily by differences in fertiliser use efficiency. The differences between fruits are partly explained by variation in primary cultivation practices, yields, and refuse generated during home preparation.

A strong correlation between the single enviro-nutritional score of the foods and NVS is consistent with the score being more heavily influenced by the NVS and less by the environmental impact, although the regression does not partition their relative contributions. The near-zero correlation for animal-source foods (R^2^ = 0.00) between the single enviro-nutritional score and the NVS (Fig. 4B) reflects the fact that both the NVS and per-kg environmental impact vary substantially—mainly due to differences in feed conversion, composition of the feed, and manure and enteric emissions and largely independently within this group: high-NVS foods are not systematically lower- or higher-impact, so NVS alone fails to predict the enviro-nutritional single score. In contrast, the stronger correlation for starchy staples (R^2^ = 0.65) arises because per-kg environmental impact is comparatively homogeneous across cereals and other staples, so the enviro-nutritional single score is dominated by variation in NVS itself, yielding a roughly linear relationship across the group's NVS range. The weak association among vegetables (R^2^ = 0.15) might reflect heterogeneity in vegetable types (eg, leafy vegetables, roots, bulbs, and fruit vegetables), their crop-management practices, and the inclusion of some by-products assigned zero cultivation impact.

Our analysis highlights that consumed food quantity can differ substantially from purchased quantity due to food waste, discarded inedible parts (refuse), and water loss or gain during cooking. When undertaking nLCA, environmental impacts must be apportioned correctly to the nutritional value of consumed food, considering mass changes during cooking and refuse. For example, durian skin comprises 76% of the fruit and becomes refuse during home preparation; this outer layer is associated with more than half of the climate change impact due to transport and end-of-life methane emissions. Inclusion of these impacts helps identify opportunities for improvement, such as skin removal prior to distribution and alternative end-of-life management.

Co-production is a feature of many agricultural systems, and this study used economic allocation with several notable exceptions.^20^ Pumpkin leaves, sweet potato leaves, tree fern, and drumstick (moringa) leaves were modelled with zero primary production impacts due to their negligible economic value as co-products. Conversely, as spent hens have little economic value in Indonesia, all primary production impacts were allocated to eggs and none to chicken meat; similarly, all impacts from fisheries were allocated to fish meat. For the green leafy vegetables, increased consumption would be limited by demand for the corresponding main crops (eg, pumpkin, sweet potato), and the same applies to chicken and beef organs as co-products—though current consumption of these co-products is only a fraction of amounts available.^27^

A limitation of our analysis is that environmental impacts were calculated for the supply chains of countries providing the largest quantity of each food item to Indonesia, and limited data availability means results are not representative of average Indonesian consumption.^15^ Additionally, we were unable to account for sub-national differences.^28^^,^^29^ Indonesian agricultural practices are highly diverse: volcanic soils in Java and Bali enable high-yield rice and vegetables, while mountainous areas benefit from distinct rainfall regimes and specialised farming practices. Future research should capture this heterogeneity through targeted data collection. For example, shrimp was modelled using proxy data from China, and future research should confirm similarities to the large domestic Indonesian shrimp industry.^30^^,^^31^ Significant investment should be directed at higher quality, sub-nationally disaggregated data, particularly for livestock management, transport distances and modes, and food losses.^22^

The results of some impact categories were more uncertain than others because of inventory data uncertainty. Water use assessment should be investigated further with region-specific characterisation factors rather than national averages.^21^ The toxicity impact categories, which contribute to the single score but are not reported individually, also had a substantial effect on the relative position of some foods. Toxicity accounted for more than half of the single score for 14 of the 90 foods (Supplementary Table S1), most notably mung bean sprouts (86%), red beans and mung beans (both 85%), and sunflower seeds from China (79%). For these foods the contribution is dominated by freshwater ecotoxicity, reflecting both reported pesticide use and comparatively low impacts in other categories owing to low fertiliser and diesel inputs relative to most other crops. Rice, potato, noodles, and pasta showed similarly high ecotoxicity contributions (52–61%), whereas for sweet peppers the toxicity contribution (51%) was almost entirely attributable to human toxicity rather than ecotoxicity. For food composition, nutrient density of the same food can show great variation depending on cultivars, production methods, soil conditions, and culinary traditions. Tailoring NVS scores to different regions and demographic groups would support calculation of more contextually relevant nLCA results.^32^^,^^33^ Beyond the nutritional and environmental dimensions assessed here, a fuller account of food system sustainability would incorporate actual health impacts as a third dimension. The NVS captures factors predictive of noncommunicable disease risk but does not quantify disease burden, and integrating epidemiological risk estimates with nutrient profiling and LCA remains an unresolved challenge identified by FAO.^11^

This type of assessment requires pre-selection of foods screened for suitability in a specific diet or additional interpretation. Screening criteria may include cultural acceptability, ethical aspects, political priorities, additional environmental criteria such as biodiversity, availability, affordability, dietary restrictions, and known adverse health outcomes. Four items in the animal-source foods and pulses, nuts, and seeds groups (beef and chicken organs, edamame, mussels, and tuna) have NVS ≥65 and rank in the top 20% least impactful foods for four or more impact categories, suggesting they might represent good candidates for local prioritisation. However, our enviro-nutritional efficiency results should be regarded as providing insights requiring further contextualisation, rather than generating oversimplified recommendations.

Future applications should include a representative list of common multi-ingredient foods relevant for the decision context (eg, nasi goreng, soto, and mie goreng in Indonesia). Congee was the only such food considered here, and its low NVS—mostly due to high salt content as street food—is indicative of the important role of meal ingredients. Analysis of complex foods requires accurate contextual data on ingredients and proportions in recipes. This extension matters because most foods are eaten as composite dishes whose nutritional value and environmental impact cannot be inferred from any single ingredient, as congee illustrates, where one added ingredient can have a large impact on the nutritional score. It is methodologically challenging because, in addition to representative recipe data, it also requires consistent apportioning of shared processing, cooking, and waste burdens across ingredients, and application of the nutrient profiling step to the assembled dish rather than to its components. These questions remain largely unresolved and are a priority for future nLCA method development for meal- and diet-level assessments.

This analysis applies a novel nLCA-specific metric to inform Indonesian policy and interventions at the nexus of nutritional and environmental performance. The results can guide incremental changes in food choices (eg, promoting green leafy vegetables, papaya, guava, cantaloupe, milk, yogurt, chicken organs, whole grains, and soy products); improvements in food preparation and waste management (eg, decreasing salt in congee, durian skin removal); alternative sourcing of foods (eg, domestically produced fruits and nuts); and improvements to supply chain performance (eg, increasing fertiliser-use and irrigation efficiency). For larger dietary shifts, these attributional results should be complemented by consequential analysis to capture the indirect substitutions and supply-chain effects such changes can trigger.

The enviro-nutritional efficiency indicator developed in this study provides succinct information to support policy-making and programmatic decisions. It can be adapted to different nutritional and environmental priorities by adjusting NVS component weightings and LCA impact category selections and tailored to different populations and contexts globally. The use of nLCA enables impacts throughout the entire food supply chain to be assessed across a range of impact categories by adopting a nutritional lens, mitigating the current tendency for carbon tunnel vision and other single-issue-based decision support. The Indonesian case study has provided proof of concept, and additional data collection, uncertainty analysis, and interpretation will confirm the feasibility of alternative future scenarios.

## Contributors

TB, FO, SJM, GAM, and TP proposed the conceptualisation of this study. TP, GAM, and SJM carried out the methodology, visualisation, and investigation. SJM, TB, FO, GAM, and TP wrote the original draft. SJM, TB, FO, GAM, TP, and RCP reviewed and edited the draft. SJM, GAM, and TP directly accessed and verified the underlying life cycle inventory and results data, and FO and TB verified the nutritional data. TB was responsible for the decision to submit the manuscript for publication.

## Data sharing statement

All data underlying this study, including the full life cycle inventory, nutritional inputs, characterisation factors, and food-level results, are openly available in the accompanying Mendeley Data repository (https://doi.org/10.17632/2fzwjty3jc.1). The Nutritional Value Score data used as the functional unit in the analysis is available on GitHub (https://github.com/GAINAlliance/NVS). No additional datasets were generated or analysed that are not included in the published article or its accompanying open-access dataset.

## Declaration of interests

SJM and TP report funding from the Global Alliance for Improved Nutrition to support their time on this manuscript. The other authors declare no competing interests.
