# Supplementary material for: Integrated environmental and nutritional efficiencies of local foods in Indonesia: a cradle-to-grave enviro-nutritional life cycle assessment
Source: Lancet Reg Health West Pac. 2026 Sep 9;74:101977. doi: 10.1016/j.lanwpc.2026.101977 (PMC13581981; doi:10.1016/j.lanwpc.2026.101977)
Supplement: Supplementary file 1 [file mmc1.docx]

**Supplementary Material**

*Integrated Environmental and Nutritional Efficiencies of Local Foods in Indonesia: a cradle-to-grave enviro-nutritional life cycle assessment*

**Supplementary Table S1**

**Table S1. Contribution of the toxicity impact categories to the Environmental Footprint 3.1 single score for 90 commonly consumed Indonesian foods.** Values are the percentage of each food’s total EF3.1 single score, expressed per nutritional functional unit (100 Nutritional Value Score), that is attributable to freshwater ecotoxicity, to human toxicity (cancer and non-cancer combined), and to the two together. The single score is calculated across the complete Environmental Footprint impact category set and is the quantity plotted in Figure 4, expressed per 100 NVS; the toxicity categories are not reported as individual midpoint indicators because of the large uncertainties in toxicity characterisation factors. Foods are listed in descending order of total toxicity contribution. Origin denotes the modelled country of primary production (“Domestic” = Indonesia).

| **Food** | **Origin** | **Freshwater ecotoxicity (%)** | **Human toxicity (%)** | **Total toxicity (%)** |
| --- | --- | --- | --- | --- |
| Mung bean sprouts | Domestic | 82.9 | 3.0 | 86.0 |
| Red beans | Myanmar | 81.6 | 3.0 | 84.6 |
| Mung beans | Myanmar | 81.6 | 3.0 | 84.6 |
| Sunflower seeds | China | 76.6 | 2.8 | 79.4 |
| Potato | Domestic | 59.7 | 4.7 | 64.4 |
| Brown rice | Domestic | 61.3 | 3.0 | 64.2 |
| White rice | Domestic | 58.2 | 2.9 | 61.1 |
| Congee (rice porridge) | Domestic | 58.2 | 2.9 | 61.1 |
| White rice noodles | Domestic | 55.4 | 2.9 | 58.4 |
| Refined wheat pasta (Australia) | Australia | 53.6 | 4.0 | 57.6 |
| Whole wheat pasta (Australia) | Australia | 51.8 | 4.1 | 55.9 |
| Whole wheat noodles (Australia) | Australia | 51.8 | 4.1 | 55.9 |
| Red pepper | Domestic | 2.3 | 49.1 | 51.4 |
| Green pepper | Domestic | 2.3 | 49.1 | 51.4 |
| Sweet corn | Domestic | 43.5 | 3.4 | 46.9 |
| Edamame | USA/domestic | 32.0 | 4.7 | 36.7 |
| Chicken | Domestic | 29.7 | 2.6 | 32.2 |
| Pork | USA | 29.6 | 2.1 | 31.8 |
| Chicken organs | Domestic | 26.3 | 3.2 | 29.5 |
| Refined wheat pasta (Ukraine) | Ukraine | 20.9 | 4.9 | 25.8 |
| Tofu | USA/domestic | 20.2 | 5.1 | 25.3 |
| Tempeh | USA/domestic | 20.1 | 5.0 | 25.0 |
| Whole wheat pasta (Ukraine) | Ukraine | 19.6 | 4.9 | 24.5 |
| Whole wheat noodles (Ukraine) | Ukraine | 19.6 | 4.9 | 24.5 |
| Egg | Domestic | 21.1 | 2.9 | 24.0 |
| Pork | Europe | 17.6 | 3.2 | 20.8 |
| Pineapple | Domestic | 7.4 | 13.0 | 20.4 |
| Coconut | Domestic | 13.1 | 5.8 | 19.0 |
| Cantaloupe | Domestic | 6.3 | 12.4 | 18.7 |
| Watermelon | Domestic | 6.3 | 12.4 | 18.7 |
| Tangerine | China | 8.9 | 9.5 | 18.4 |
| Pumpkin leaves | Domestic | 5.8 | 12.1 | 17.8 |
| Drumstick leaves | Domestic | 5.8 | 12.1 | 17.8 |
| Sweet potato leaves | Domestic | 5.8 | 12.1 | 17.8 |
| Banana | Domestic | 8.3 | 9.3 | 17.6 |
| Papaya | Domestic | 5.6 | 11.5 | 17.1 |
| Pear | China | 5.8 | 11.0 | 16.8 |
| Apple | China | 6.5 | 10.1 | 16.7 |
| Tree fern | Domestic | 5.2 | 11.3 | 16.5 |
| Carrots | Domestic | 5.1 | 11.2 | 16.3 |
| Grapefruit | China | 13.1 | 2.9 | 16.0 |
| Peanut butter | Domestic | 8.9 | 6.3 | 15.1 |
| Guava | Domestic | 3.5 | 11.6 | 15.1 |
| Peanuts | Domestic | 8.8 | 6.2 | 14.9 |
| Mussels | Domestic | 4.8 | 8.6 | 13.4 |
| Durian | Domestic | 4.5 | 8.5 | 13.0 |
| Orange | Domestic | 4.9 | 7.0 | 11.9 |
| Cucumber | Domestic | 4.5 | 7.4 | 11.9 |
| Chayote | Domestic | 4.0 | 7.8 | 11.8 |
| Plain whole yogurt (France) | France | 6.0 | 5.7 | 11.7 |
| Shrimps | Domestic | 4.3 | 7.1 | 11.5 |
| Unsweetened soymilk | USA/domestic | 5.1 | 6.2 | 11.3 |
| Longans | Domestic | 3.7 | 7.1 | 10.8 |
| Mango | Domestic | 3.5 | 6.9 | 10.4 |
| Radish | Domestic | 3.8 | 5.8 | 9.5 |
| Avocado | Domestic | 2.4 | 7.0 | 9.4 |
| Cabbage | Domestic | 3.6 | 5.6 | 9.2 |
| Chinese cabbage | Domestic | 3.6 | 5.0 | 8.7 |
| Water spinach | Domestic | 3.1 | 5.4 | 8.5 |
| Pumpkin | Domestic | 4.2 | 4.2 | 8.4 |
| Starfruit | Domestic | 2.7 | 5.5 | 8.2 |
| Mushrooms | Domestic | 2.6 | 5.4 | 8.0 |
| Eggplant | Domestic | 3.0 | 4.7 | 7.7 |
| Plain whole yogurt (domestic) | Domestic | 4.1 | 3.4 | 7.5 |
| Rabbit | Domestic | 3.1 | 4.3 | 7.4 |
| Long bean | Domestic | 3.0 | 4.1 | 7.1 |
| Zucchini | Domestic | 4.1 | 2.9 | 7.0 |
| Cauliflower | Domestic | 2.9 | 4.1 | 6.9 |
| Chinese broccoli | Domestic | 3.0 | 3.7 | 6.7 |
| Broccoli | Domestic | 3.0 | 3.7 | 6.7 |
| Tomatoes | Domestic | 2.6 | 4.0 | 6.6 |
| Luffa gourd | Domestic | 3.5 | 3.0 | 6.5 |
| Bitter melon | Domestic | 3.5 | 3.0 | 6.5 |
| Whole goat milk | Domestic | 2.2 | 3.3 | 5.5 |
| Tuna | Domestic | 1.5 | 3.8 | 5.3 |
| Cashews | Vietnam | 1.1 | 4.1 | 5.2 |
| Spinach | Domestic | 2.5 | 2.7 | 5.2 |
| Whole sheep milk | Domestic | 1.4 | 3.1 | 4.6 |
| Tilapia | Domestic | 2.9 | 1.6 | 4.5 |
| Whole cow milk | Domestic | 1.7 | 2.6 | 4.3 |
| Green beans | Domestic | 2.0 | 2.1 | 4.1 |
| Winged beans | Domestic | 2.0 | 2.1 | 4.1 |
| Beef organs (Australia) | Australia | 1.0 | 2.6 | 3.6 |
| Low-fat cottage cheese | New Zealand | 1.0 | 2.0 | 2.9 |
| Whole milk powder | New Zealand | 0.9 | 1.8 | 2.8 |
| Beef organs (India) | India | 0.9 | 1.8 | 2.7 |
| Full-fat cheese | New Zealand | 0.9 | 1.8 | 2.7 |
| Beef (Australia) | Australia | 0.7 | 1.7 | 2.4 |
| Lamb | Domestic | 0.8 | 1.4 | 2.1 |
| Beef (India) | India | 0.7 | 1.3 | 1.9 |
